# Epidemiological Characteristics and Clinical Impact of Macrolide‐Resistant *Mycoplasma pneumoniae* Infection in Children in Southwest China, 2021–2025

**DOI:** 10.1111/crj.70233

**Published:** 2026-09-30

**Authors:** Ting Liu, Sha‐sha Chen, Xiao‐lin Chen, Si‐yu Tan, Qin‐xue Lu, Yan Zhou

**Affiliations:** ^1^ Department of Laboratory Medicine West China Second University Hospital, Sichuan University Chengdu China; ^2^ Key Laboratory of Obstetric & Gynecologic and Pediatric Diseases and Birth Defects of Ministry of Education Sichuan University Chengdu China; ^3^ State Key Laboratory of Biotherapy and Cancer Center/National Collaborative Innovation Center for Biotherapy Sichuan University Chengdu China

**Keywords:** children, clinical outcomes, epidemiology, macrolide resistance, *mycoplasma pneumoniae*

## Abstract

**Background:**

*Mycoplasma pneumoniae* (MP) is a key pathogen causing pediatric community‐acquired pneumonia. Increasing macrolide resistance poses considerable clinical challenges. This study investigated MP epidemiology and macrolide resistance in children aged 0–14 years in Southwest China from 2021–2025.

**Methods:**

Retrospective analysis of MP antibody, nucleic acid, and resistance gene detection data. MP infections were categorized into macrolide‐resistant (MRMP) and macrolide‐sensitive (MSMP) groups. Clinical information was collected and compared.

**Results:**

Positive rates for MP‐RNA, DNA, IgM, and total antibodies were 12.43%, 9.82%, 30.19%, and 49.82%, respectively. Infections predominantly affected children aged 2–8 years. The macrolide resistance rate was 87.87%. Compared with the MSMP group, the MRMP group had longer hospital stays (+0.74 days), higher rates of fiberoptic bronchoscopy (+19.6%), and a significantly higher initial macrolide treatment failure rate, leading to increased doxycycline use (+20.8%).

**Conclusion:**

MRMP infections are highly prevalent in Southwest China and are associated with more complex clinical conditions and worse outcomes. Early resistance gene detection is recommended for hospitalized children with suspected MP infection during epidemic seasons. For children aged 2–8 years with persistent high fever and imaging findings of lung consolidation or mucus plugs, high suspicion of MRMP is warranted, and timely fiberoptic bronchoscopy should be considered.

## Graphical abstract

This study reveals a persistently high macrolide resistance rate (87.87%) in *Mycoplasma pneumoniae* among children in Southwest China (2021–2025), predominantly affecting those aged 2–8 years. MRMP infections are associated with longer hospital stays, increased bronchoscopy use, and higher doxycycline prescriptions, underscoring the urgent need for resistance‐guided therapy and heightened clinical vigilance.

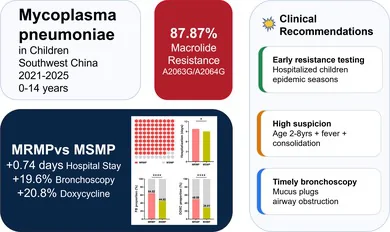

**Abbreviations**
CAP: community‐acquired pneumonia
Ct: cycle threshold
HIS: hospital information system
MP:
*mycoplasma pneumoniae*

MPP:
*mycoplasma pneumoniae* pneumonia
MRMP: macrolide‐resistant *mycoplasma pneumoniae*

MSMP: macrolide‐sensitive *mycoplasma pneumoniae*

RLUs: relative light units
S/CO: sample‐to‐cutoff ratio

## 1 Introduction

*Mycoplasma pneumoniae* (MP) is a major pathogen of community‐acquired pneumonia (CAP) in children and currently accounts for 40% of pneumonia cases among respiratory infections, highlighting its importance for child health [1, 2]. MP is transmitted between individuals through respiratory droplets produced by coughing, and outbreaks often occur in kindergartens, elementary schools, and middle schools [3]. MP infections can occur sporadically throughout the year or lead to widespread community outbreaks. Infections typically present as upper respiratory infections in children under 3 years of age, whereas those over 5 years of age often exhibit bronchitis and pneumonia [4, 5]. Although most children infected with MP can recover on their own, some younger children may develop severe *mycoplasma pneumoniae* pneumonia (MPP) due to their underdeveloped immune systems [6]. Severe MPP can manifest as lung consolidation, inflammatory storms, encephalitis, and even respiratory failure, posing a life‐threatening risk [7]. Additionally, some children may fail to respond to appropriate treatment and subsequently develop refractory MPP, resulting in worsened disease progression, extended illness, and increased treatment costs [8]. Given the substantial disease burden and potential for severe outcomes, understanding the factors that complicate MP infection, particularly antimicrobial resistance, has become increasingly critical.

In recent years, the increasing incidence of respiratory infections in children, along with the increasing frequency and scope of antibiotic use, has led to a significant increase in MP resistance to macrolide antibiotics [9, 10]. Because the first isolation of macrolide‐resistant MP (MRMP) strains from pediatric pneumonia patients in Japan in 2000, Asian countries have become the primary regions for the emergence of MRMP. The macrolide resistance rate of MP in South Korea was reported to be as high as 78.5% between 2019 and 2020 [11], whereas in northern China, the resistance rate exceeded 90% from 2015 to 2020 [12]. In Southwest China, recent data have become available. A nationwide genomic surveillance study conducted during the 2023–2024 resurgence reported that 96.31% of MP isolates carried macrolide resistance mutations [13]. Specifically in Chengdu, a single‐center study of hospitalized children with MP pneumonia found that the macrolide resistance gene positivity rate reached 97.3% in 2024 [14], confirming an exceptionally high resistance burden in this region. The resistance of MP is closely related to specific genetic mutations, particularly the A2063G and/or A2064G mutations in 23S rRNA, which significantly increase the minimum inhibitory concentration of macrolide antibiotics [15]. These alarming resistance rates underscore the urgent need to translate resistance detection into actionable clinical strategies, yet several critical gaps remain in this translation.

In this context, three major clinical challenges have emerged. First, although macrolide antibiotics are the first‐line therapy for MP infections, the likelihood of clinical improvement remains low for some patients with MRMP, even after standard treatment [16]; however, there is currently a lack of studies on the association between macrolide resistance and clinical severity, making it difficult to provide accurate prognostic predictions. Second, although alternative second‐line medications, such as tetracyclines (doxycycline or minocycline) and fluoroquinolones (ciprofloxacin or levofloxacin), may improve clinical symptoms in some children after macrolide treatment failure [17], there is a lack of clear evidence regarding the optimal timing for initiating second‐line therapy, as well as appropriate dosage and treatment duration in pediatric patients [17]. Third, epidemiological data on MP infection in children in Southwest China remain scarce, particularly regarding infection and resistance patterns during the postpandemic period following the restoration of social order. Addressing these gaps is essential for optimizing clinical management.

Recent research on MP in China has mainly focused on trends observed before and during the COVID‐19 pandemic [18, 19, 20]. However, these studies are largely based on data collected during the implementation of nonpharmaceutical interventions (such as mask‐wearing and social distancing) and lack analysis of data following the restoration of social order postpandemic. Therefore, this study aims to conduct a retrospective analysis of MP antibody, nucleic acid, and resistance gene testing data from children in Southwest China between 2021 and 2025. Specifically, we seek to (1) characterize the epidemiological features, including infection rate, macrolide resistance rate, and age distribution of MP infection in this region during the postpandemic period, addressing the current data gap in Southwest China; (2) investigate the association between macrolide resistance and clinical severity by comparing clinical manifestations, proportion of patients undergoing fiberoptic bronchoscopy, and hospitalization outcomes between resistant and nonresistant groups; and (3) describe real world treatment patterns and outcomes in children with resistant MP infection, providing evidence to inform the appropriate use of second‐line antibiotics. Collectively, these findings aim to provide laboratory‐based evidence to support accurate diagnosis, optimize treatment strategies, and improve clinical management of MP infection in pediatric patients.

## 2 Methods

### 2.1 Patients and Data Collection

This retrospective study included children with suspected respiratory infections who underwent MP antibody testing (total antibodies and IgM), nucleic acid testing (DNA and RNA), and drug resistance gene testing (A2063G and/or A2064G mutations) at a large, university‐affiliated tertiary care hospital in China between January 2021 and December 2025. The inclusion criteria were as follows: (1) aged ≤ 14 years; (2) absence of immunodeficiency; (3) no congenital organ dysfunction or hematologic disorders; and (4) no other concurrent infectious diseases. Clinical data, including age, sex, length of hospital stay, the presence of bacterial coinfections, use of fiberoptic bronchoscopy, and treatment with doxycycline, were retrospectively collected from the hospital information system (HIS). This study was approved by the Medical Ethics Committee of West China Second University Hospital, Sichuan University (No. 2025001). Broad informed consent for the use of clinical data in research was obtained from all patients and their parents at admission. As this was a retrospective study analyzing deidentified clinical data, the ethics committee waived the requirement for additional informed consent.

### 2.2 MP Total Antibodies Detection

Total antibodies against MP in serum samples were detected using a passive agglutination method. The principle involves coating MP cell membrane components onto gelatin particles to form sensitized particles; agglutination occurs when these particles react with MP antibodies in the serum. The test was performed according to the manufacturer's instructions (Zhuhai Livzon Diagnostics Inc., Zhuhai, Guangdong, China). Briefly, particles were reconstituted and serially diluted to determine antibody titers, with titers ≥ 1:160 considered positive. Negative and positive controls were included in each test to ensure result reliability.

### 2.3 MP‐IgM Detection

Specific IgM antibodies in serum samples were detected using an indirect chemiluminescence assay. In this method, magnetic microspheres coated with MP antigens bind to horseradish peroxidase‐labeled human IgM antibodies, forming a complex that catalyzes a luminescent substrate and generates a light signal proportional to the IgM antibody content in the sample. The procedure was carried out according to the instructions of the reagent kit (Autobio Biochemistry Co. Ltd., Zhengzhou, Henan, China) and included sample preparation, reagent loading, incubation at 37°C for 17 min, washing, and luminescence detection. A sample‐to‐cutoff ratio (S/CO) ≥ 1.00 was considered positive. Quality control was maintained with a coefficient of variation below 10%.

### 2.4 MP‐DNA Detection

MP‐DNA was detected using real‐time PCR with an ABI 7500 PCR system (Thermo Fisher Scientific Co. Ltd., Waltham, Massachusetts, USA) and commercially available detection kits (Xiamen Amplly Biotechnology Co. Ltd., Xiamen, Fujian, China). Throat swab samples were preserved in 0.5 mL of sterile saline, and nucleic acid extraction was performed using specialized reagents. The reaction system was configured according to the kit instructions, with amplification conditions set as follows: 38°C for 5 min, followed by 95°C for 2 min, then 40 cycles of 95°C for 15 s and 58 °C for 50 s. Fluorescence signals were collected at 58°C. The detection sensitivity was 5.0 × 10^2^ copies/mL, and samples with a cycle threshold (Ct) value ≤ 38 were considered positive.

### 2.5 MP‐RNA Detection

MP‐RNA was detected using a seven‐pathogen respiratory nucleic acid detection kit (Wuhan Zhongzhi Biotechnology Co. Ltd., Wuhan, Hubei, China). Throat swab samples were preserved in 1 mL of phosphate‐buffered saline or saline. Following cell lysis buffer treatment, T7 nucleic acid amplification technology was used for specific RNA amplification on an ABI 7500 PCR system (Thermo Fisher Scientific Co. Ltd., Waltham, Massachusetts, USA). The amplification products hybridized with specific probes and were detected using biotin signal amplification. The detection process included sample lysis, nucleic acid amplification, hybridization, signal amplification, and chemiluminescent detection. Results were quantified in relative light units (RLUs) using a chemiluminescence instrument, with an RLU ratio > 1.0 considered positive. Each test included one positive control, two negative controls, and an 18S rRNA internal control probe to ensure result reliability.

### 2.6 Definitions

Active MP infection was defined as a positive MP‐DNA or MP‐RNA result from throat swabs, or a positive MP‐IgM result (S/CO ≥ 1.00) in patients with acute respiratory symptoms. Positive total antibodies alone (titers ≥ 1:160), without nucleic acid or IgM positivity, were considered indicative of prior exposure rather than active infection and were not used as a criterion for active disease. Initial macrolide treatment failure was defined as persistence of fever (≥ 38.0°C) for ≥ 72 h after initiation of macrolide therapy, with or without worsening of respiratory symptoms or radiographic findings, prompting a change to second‐line antibiotics (doxycycline or levofloxacin).

### 2.7 Macrolide Resistance Gene Detection

Macrolide resistance mutations (A2063G and/or A2064G) in MP were detected using fluorescence PCR on an ABI 7500 PCR system (Thermo Fisher Scientific Co. Ltd., Waltham, Massachusetts, USA) from throat swab samples. TaqMan fluorescence probe technology was used to design primers and probes targeting the MP P1 gene (labeled with VIC) and the 23S rRNA gene mutations A2063G and A2064G (labeled with FAM). The reaction system was prepared according to the kit instructions (Jiangsu Mole Bioscience Co. Ltd., Taizhou, Jiangsu, China), and PCR conditions were as follows: 50°C for 2 min and 95°C for 2 min, followed by 40 cycles of 95°C for 15 s and 64°C for 60 s. Fluorescence signals were collected at 64°C. Results were interpreted based on Ct values: A VIC Ct < 35.01 indicated MP positive, and a FAM Ct < 35.33 indicated the presence of either the A2063G or A2064G mutation. Samples positive for either mutation were classified as genotypically MRMP, and those negative for both as genotypically MSMP. Of note, genotypic resistance does not necessarily equate to clinical phenotypic resistance; treatment response was therefore assessed based on clinical manifestations and antibiotic use, rather than genetic results alone. The kit also included a CY5‐labeled internal control probe and utilized strong positive, weak positive, and negative controls to ensure reliability and accuracy.

### 2.8 Statistical Analysis

Descriptive statistics were presented as frequencies and percentages. The chi‐square test was used to compare the clinical characteristics between the macrolide‐resistant MP (MRMP) and macrolide‐sensitive MP (MSMP) groups. A two‐sided *p*‐value < 0.05 was considered statistically significant. All statistical analyses and graphical presentations were performed using IBM SPSS Statistics for Windows version 20.0 (IBM Corp., Armonk, NY, USA) and GraphPad Prism 6.0 (GraphPad Software, San Diego, CA, USA).

## 3 Results

### 3.1 Positive Rates, Annual Trends, and Age Distribution of MP Nucleic Acid and Antibody Tests in Children

From 2021 to 2025, a total of children aged 0–14 years who underwent testing for MP nucleic acid (MP‐RNA and MP‐DNA) and antibodies (MP‐IgM and total antibodies) were enrolled. The overall positive rates were 12.43% (138/1110) for MP‐RNA, 9.82% (8647/88076) for MP‐DNA, 30.19% (20 698/68551) for MP‐IgM, and 49.82% (34 154/68551) for total antibodies, indicating a high burden of MP infection in children. The relatively high total antibody positivity suggests that a substantial proportion of children had prior MP exposure (Figure 1A). Over the 5‐year period from 2021 to 2025, the annual positivity rates of all four testing methods showed a fluctuating upward trend, with a notable decline in DNA and RNA positivity rates in 2025, consistent with the epidemiological patterns of respiratory infections. In particular, the MP‐IgM positive rate increased progressively year by year: 12.24% (1335/10905) in 2021, 24.00% (3094/12891) in 2022, 29.74% (6928/23298) in 2023, 43.12% (5723/13273) in 2024, and 44.21% (3618/8184) in 2025, reflecting a rising prevalence and potentially increasing severity of MP infections in recent years (Figure 1B). Age distribution analysis showed that MP antibody‐positive cases were most frequent in children aged 2–4 years, whereas nucleic acid‐positive cases were mainly distributed in the 2–4 years and 6–8 years age groups. These findings indicate that preschool and early school‐age children, particularly those aged 2–8 years, represent the primary population affected by MP infections (Figure 1C).

**FIGURE 1:**
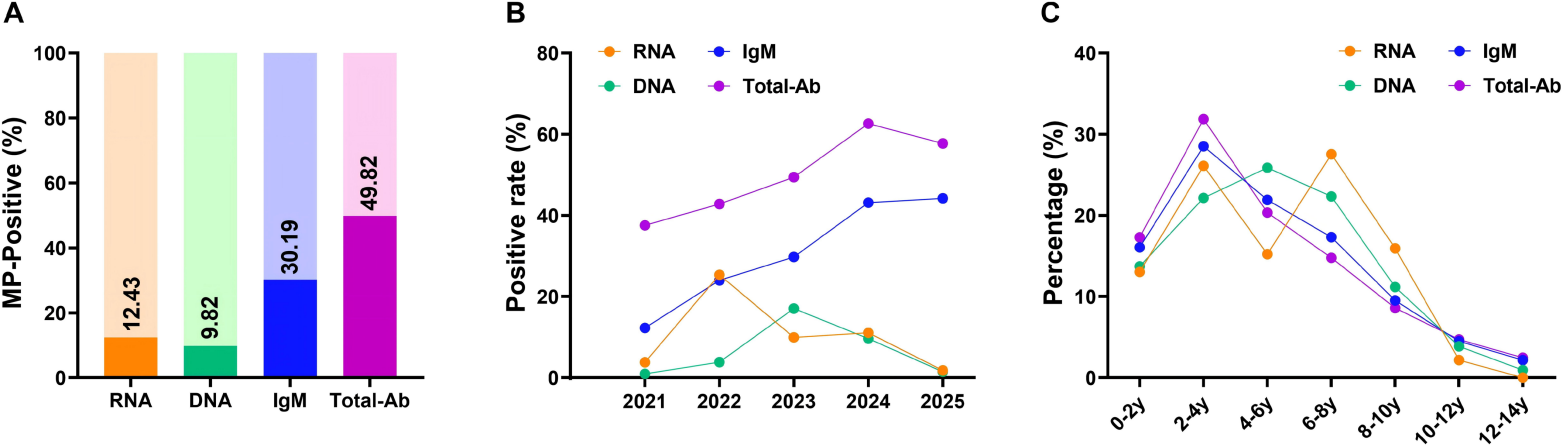
Positive rates, annual trends, and age distribution of MP nucleic acid and antibody tests in children from 2021 to 2025. (A) Overall positive rates of MP‐RNA, MP‐DNA, MP‐IgM, and total antibodies. Total antibody positivity was highest (49.82%), indicating a high burden of prior MP exposure in children. (B) Annual positivity trends of MP‐RNA, MP‐DNA, MP‐IgM, and total antibodies from 2021 to 2025. All four methods showed a fluctuating upward trend over the 5‐year period, with a notable decline in DNA and RNA positivity rates in 2025, consistent with epidemiological patterns of respiratory infections. Notably, MP‐IgM positivity increased progressively from 12.24% in 2021 to 44.21% in 2025. (C) Age distribution of MP antibody‐positive and nucleic acid‐positive cases. Antibody‐positive cases peaked in children aged 2–4 years, whereas nucleic acid‐positive cases were mainly distributed in the 2–4 years and 6–8 years age groups, indicating that children aged 2–8 years are the primary population affected by MP infections.

### 3.2 Macrolide Resistance Rate and Clinical Characteristics of MRMP Versus MSMP Infections

From 2022–2025, macrolide resistance testing was performed on 25,209 MP‐positive patients (testing was not conducted in 2021). MRMP was defined by the presence of A2063G and/or A2064G mutations detected via real‐time PCR, whereas MSMP was defined by the absence of these mutations. The overall MRMP positivity rate was 87.87% (22,152/25,209) (Figure 2A), with annual rates of 88.14% (52/59) in 2022, 85.98% (9,945/11,566) in 2023, 89.50% (11,785/13,167) in 2024, and 88.73% (370/417) in 2025 (Figure 2B), indicating that macrolide‐resistant MP has become a major issue in pediatric infections in recent years. Age and sex distributions were similar between the MRMP and MSMP groups (Figure 2C,D), and no significant difference was observed in hospitalization rates (4.63% [818/17649] vs. 4.97% [126/2535]) (Figure 2E). After excluding patients with incomplete clinical data, 730 hospitalized MRMP patients and 118 hospitalized MSMP patients were included for further analysis (Figures 2F–J). The average length of hospital stay was significantly longer in the MRMP group than in the MSMP group (9.79 ± 0.19 vs. 9.05 ± 0.36 days, *p* = 0.0351) (Figure 2F). Coinfection patterns were then evaluated in detail. Overall coinfection rates were similar between the two groups (21.92% [160/730] vs. 22.88% [27/118]) (Figure 2G). However, stratification by pathogen type revealed distinct profiles (Figure 2J), the MRMP group had a significantly higher proportion of pure bacterial coinfections (7.95% [58/730] vs. 2.54% [3/118], *p* = 0.0439), whereas the MSMP group exhibited a significantly higher proportion of pure viral coinfections (16.95% [20/118] vs. 10.68% [78/730], *p* = 0.0336). Mixed bacterial–viral and fungal coinfections were rare and did not differ significantly between groups. The proportion of patients undergoing fiberoptic bronchoscopy was significantly higher in the MRMP group (64.52%, 471/730) than in the MSMP group (44.92%, 53/118, *p* < 0.0001) (Figure 2H). In addition, the MRMP group had a significantly higher initial macrolide treatment failure rate, which led to subsequent increased use of doxycycline (49.59% [362/730] vs. 28.81% [34/118], +20.8%, *p* < 0.0001) (Figure 2I). To adjust for potential confounders, multivariate regression analyses were performed. After adjusting for age, sex, and coinfection, MRMP infection remained independently associated with prolonged hospital stay (*β* = 1.12, 95% CI: 0.61–1.63, *p* < 0.0001). Multivariate logistic regression analyses, adjusted for the same covariates, further confirmed that MRMP infection was independently associated with higher rates of fiberoptic bronchoscopy (adjusted OR = 8.84, 95% CI: 4.71–16.58, *p* < 0.0001) and increased doxycycline use (adjusted OR = 2.46, 95% CI: 1.70–3.56, *p* < 0.0001). Collectively, although MRMP infection was not associated with patient age or sex, it was associated with more severe clinical manifestations, prolonged illness, and increased treatment difficulty.

**FIGURE 2:**
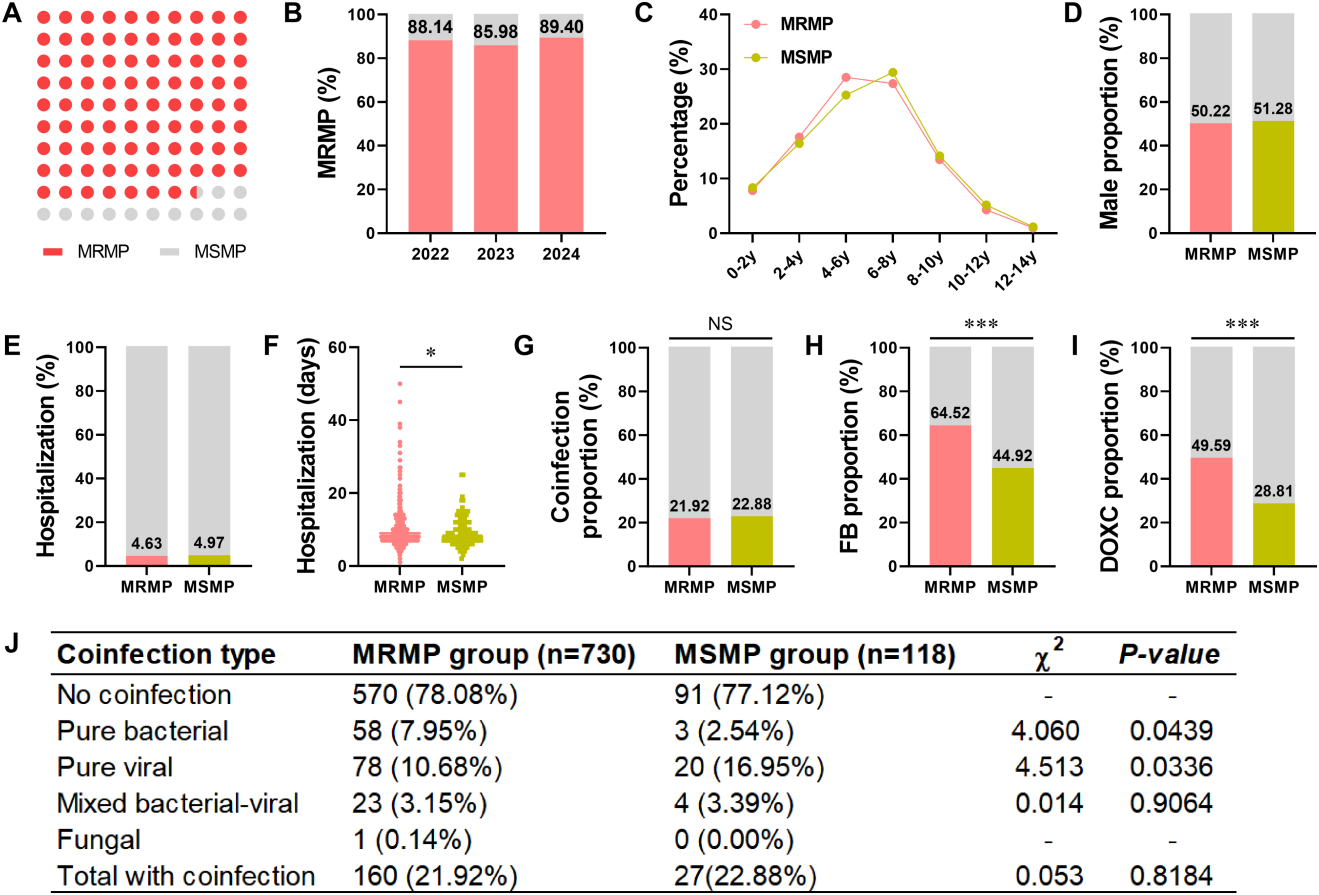
Comparison of clinical characteristics between MRMP and MSMP infections. (A) Overall MRMP positive rate across the study period. (B) Annual MRMP positive rates from 2022 to 2025. (C) Age distribution of patients with MRMP and MSMP infections, showing similar patterns between the two groups. (D) Proportion of male patients in the MRMP and MSMP groups. (E) Hospitalization rates for MRMP and MSMP infections. (F) Average length of hospital stay for hospitalized patients with MRMP and MSMP infections, compared using the Mann–Whitney *U* test. (G) Coinfection rates among hospitalized patients with MRMP and MSMP infections. (H) Proportion of hospitalized patients undergoing fiberoptic bronchoscopy in the MRMP and MSMP groups. (I) Proportion of hospitalized patients receiving doxycycline treatment in the MRMP and MSMP groups. (J) Coinfection profiles in MRMP and MSMP groups, including pure bacterial, pure viral, mixed bacterial–viral, and fungal coinfections. Data were compared using the chi‐square test unless otherwise specified. *p < 0.05, **p < 0.01, and ***p < 0.001. NS, not significant.

## 4 Discussion

Accumulated epidemiological data indicate that MP outbreaks, which previously occurred every 3–7 years, have become more frequent and prolonged over the past two decades [21, 22, 23]. Following the onset of the COVID‐19 pandemic, MP incidence declined sharply due to nonpharmaceutical interventions [22]; however, since April 2023, many countries have reported resurgent MP pneumonia cases, particularly in Asia and Europe [24, 25, 26]. Our study confirms a sustained increase in MP prevalence in Southwest China from 2021 to 2024, with the MP‐IgM positive rate rising to 43.12% in 2024, and the total antibody detection rate reaching 62.61%, indicating that over half of pediatric patients had prior MP exposure. The macrolide resistance rate remained persistently high (87.87% overall, reaching 89.50% by 2024), aligning with trends in the Western Pacific region [27]. Notably, MP infections predominantly affected children aged 2–8 years, with increasing cases in those under 3 years—a pattern diverging from earlier reports that emphasized children over 5 years [24, 28, 29]. This shift underscores the need for targeted prevention strategies for preschool‐aged children, who are particularly vulnerable due to immature immunity and kindergarten exposure.

MRMP infections impose a significantly greater clinical burden than MSMP infections [17]. Our study provides quantitative evidence across multiple dimensions: MRMP‐infected children experienced prolonged hospital stays (9.79 vs. 9.05 days, +0.74 days) and higher rates of fiberoptic bronchoscopy (64.52% vs. 44.92%, +19.6%). Furthermore, the significantly higher initial macrolide treatment failure rate in the MRMP group directly drove the increased use of doxycycline (49.59% vs. 28.81%, +20.8%), demonstrating that resistance is associated with clinical ineffectiveness of first‐line therapy. The higher proportion of bronchoscopic interventions in the MRMP group suggests that these patients are more likely to develop severe complications such as lung consolidation or mucus plugs [30], consistent with the pathophysiology of refractory MPP [9, 17]. Although the coinfection rate in our study (11.10%) was lower than that reported in South Korea (88.49%) [31], this discrepancy may be attributed to the fact that our study focused only on bacterial coinfections and the low sensitivity of the culture methods employed. Collectively, these findings suggest that MRMP infections are associated with compromised antibiotic efficacy and with greater disease severity and healthcare resource utilization.

Macrolide resistance in MP is primarily mediated by mutations at positions 2063, 2064, 2067, and 2617 in domain V of 23S rRNA, with A2063G and A2064G conferring high‐level resistance [32, 33, 34]. Our study confirms that these mutations are highly prevalent in Southwest China, and their detection provides actionable clinical information. Although tetracyclines and fluoroquinolones serve as effective second‐line options [35], their use is associated with potential adverse effects, including gastrointestinal discomfort, tooth discoloration, and musculoskeletal issues [36]. Therefore, confirming resistance before prescribing these agents is critical for balancing treatment efficacy and safety [37]. Our data show that doxycycline use was significantly higher in the MRMP group (49.59% vs. 28.81%), indicating that clinicians appropriately escalated therapy when resistance was present. However, the correlation between resistance genotype and clinical severity remains incompletely understood, as some resistant strains may still respond to macrolides due to individual variability or low‐level resistance [38, 39]. Nevertheless, timely resistance gene detection facilitates informed clinical decision‐making, enables effective communication with guardians, and supports public health surveillance of resistance trends.

Based on these findings, we propose two actionable recommendations to enhance clinical management. First, during MP epidemic seasons, hospitalized children with suspected MP infection should undergo prioritized resistance gene testing (A2063G/A2064G). For those with positive results, early consideration of second‐line therapy is recommended to avoid prolonged ineffective treatment. However, in the absence of resistance testing, MRMP should not be assumed based on clinical presentation alone, and treatment decisions should be guided by laboratory confirmation whenever possible. Second, for children aged 2–8 years presenting with persistent high fever and imaging findings suggestive of lung consolidation or mucus plugs, a high index of suspicion for MRMP infection is warranted to prompt timely resistance testing. Clinical features alone are insufficient for diagnosis, and laboratory confirmation remains essential before treatment escalation. Clinicians should be aware that fiberoptic bronchoscopy is an invasive procedure; its benefits and risks must be carefully weighed on a case‐by‐case basis, and it should be reserved for patients with persistent or progressive disease despite optimal medical therapy. Timely evaluation for bronchoscopic intervention should be pursued to address potential airway obstruction and mitigate disease progression. These recommendations are intended for hospitalized children during epidemic seasons and should be applied with caution in outpatient settings or nonepidemic periods.

Several limitations should be acknowledged. First, the retrospective design and reliance on a single‐center HIS, with the majority of our clinical analyses derived from hospitalized patients, limited the availability of additional clinical data, such as MP culture identification or genetic typing, and may restrict the generalizability of our findings to outpatients or other regions of Southwest China. Second, selection bias may be present, as the study population consisted of patients who actively sought testing, potentially overestimating positive rates. Third, PCR testing cannot differentiate active infection from carrier status, whereas serological testing may be affected by the window period and individual immune response variability. Fourth, macrolide resistance was determined genotypically by detecting A2063G/A2064G mutations via PCR, rather than phenotypically by culture‐based antimicrobial susceptibility testing. Although these mutations are well‐established markers of high‐level macrolide resistance, we acknowledge the lack of phenotypic verification. Fifth, given the retrospective nature of this study, the observed associations between MRMP infection and adverse clinical outcomes should be interpreted as correlations rather than causal relationships, and residual confounding cannot be completely excluded. Future multicenter prospective studies integrating multiple testing modalities with clinical presentation are needed to validate resistance‐guided treatment strategies.

## 5 Conclusion

In conclusion, this study demonstrates that the resurgence of *M. pneumoniae* in Southwest China from 2021 to 2025 is characterized by persistently high macrolide resistance (87.87%) and predominance in preschool‐aged children. Critically, we have quantified the clinical outcomes associated consequences of resistance: MRMP infections are associated with longer hospital stays, higher rates of fiberoptic bronchoscopy, and increased second‐line antibiotic use, thereby highlighting resistance as an important factor associated with disease severity and healthcare burden. These findings have important clinical and public health implications. First, they support a shift from empirical macrolide therapy toward resistance‐guided treatment strategies during epidemic seasons. Second, they highlight the need for heightened suspicion in children aged 2–8 years with characteristic imaging findings, even before resistance testing results are available. Third, the persistently high resistance rates underscore the urgency of strengthening regional surveillance systems to monitor resistance trends and inform antibiotic stewardship policies. Ultimately, addressing the challenge of MRMP requires integrating rapid resistance testing into clinical workflows, establishing evidence‐based algorithms for second‐line therapy, and maintaining robust surveillance to bridge the gap between laboratory detection and clinical action; thereby optimizing clinical outcomes and mitigating the threat of antimicrobial resistance in pediatric respiratory infections.

### Author Contributors

Ting Liu and Yan Zhou conceived and designed the study. Sha‐sha Chen, Xiao‐lin Chen, Si‐yu Tan, and Qin‐xue Lu performed the data collection. Ting Liu conducted the data analysis and interpretation and drafted the manuscript. Yan Zhou critically revised the manuscript for important intellectual content and supervised the study. All authors reviewed and approved the final manuscript.

## Funding

This work was supported by a Horizontal Project of Sichuan University (Grant No. 23H0345).

## Ethics Statement

This study was approved by the Medical Ethics Committee of West China Second University Hospital, Sichuan University (Approval No. 2025001).

## Conflicts of Interest

The authors declare no conflicts of interest.

## Data Availability

All data generated or analyzed during this study are included in this published article.
